# A Systematic Review and Meta-analysis of Efficacy and Safety of Mavacamten for the Treatment of Hypertrophic Cardiomyopathy

**DOI:** 10.31083/j.rcm2510375

**Published:** 2024-10-23

**Authors:** Li Zheng, Xiaotong Gu, Yumiao Chen, Deping Liu

**Affiliations:** ^1^Department of Pharmacy, China Aerospace Science & Industry Corporation 731 Hospital, 100074 Beijing, China; ^2^Department of Medical, China Aerospace Science & Industry Corporation 731 Hospital, 100074 Beijing, China; ^3^Department of Cardiovascular Medicine, Beijing Hospital, National Center of Gerontology, Institute of Geriatric Medicine, Chinese Academy of Medical Sciences, 100730 Beijing, China

**Keywords:** hypertrophic cardiomyopathy, mavacamten, meta-analysis, cardiac myosin inhibitor

## Abstract

**Background:**

Hypertrophic cardiomyopathy (HCM) is a common hereditary cardiomyopathy. Mavacamten, a first-in-class cardiac myosin inhibitor, is considered to be a specific drug for the treatment of HCM. This meta-analysis aimed to assess the efficacy and safety of mavacamten in patients with HCM.

**Methods:**

PubMed, Cochrane Library, Embase and Clinical Trials.gov databases were searched from inception to February 6, 2024 for randomized controlled trials (RCTs) which compared the efficacy and safety between mavacamten and placebo in treating HCM.

**Results:**

Six RCTs involving 732 patients were included in this meta-analysis. This meta-analysis showed that mavacamten improved the New York Heart Association (NYHA) function class [risk ratios (RR): 2.21, 95% confidence interval (CI): 1.48 to 3.30, *p* = 0.00001], Clinical Summary Score of the Kansas City Cardiomyopathy Questionnaire (KCCQ-CSS) scores [mean difference (MD): 9.33, 95% CI: 7.09 to 11.57, *p* < 0.00001] and composite functional end point (RR: 1.86, 95% CI: 1.25 to 2.78, *p* = 0.002). Meanwhile, mavacamten decreased N-terminal pro-B-type natriuretic peptide (NT-proBNP) (MD: –492.28, 95% CI: –611.55 to –373.02, *p* < 0.00001), cardiac troponin I (cTnI) (MD: –14.58, 95% CI: –26.98 to –2.17, *p* = 0.02) and Valsalva left ventricular outflow tract (LVOT) gradient (MD: –57.96, 95% CI: –82.15 to –33.78, *p* < 0.00001). The results for the incidence of ≥1 total emergent adverse event (TEAE) and ≥1 serious adverse event (SAE) showed that there was no significant difference between both groups (RR: 1.9, 95% CI: 0.97 to 1.24, *p* = 0.16) (RR: 1.06, 95% CI: 0.46 to 2.44, *p* = 0.90).

**Conclusions:**

Mavacamten has great efficacy for the treatment of HCM. Meanwhile, mavacamten did not increase the incidence of adverse events or serious adverse events.

## 1. Introduction

Hypertrophic cardiomyopathy (HCM) is considered an autosomal dominant genetic disease caused by a sarcomere protein gene mutation, which is the most common hereditary cardiac condition featuring disorganized architecture of the myocardium and left ventricular hypertrophy [1, 2, 3]. The prevalence of HCM is 0.2% in the general population, and HCM is the primary cardiac cause of sudden cardiac death [4].

The pathogenic mechanism of HCM is highly complex, and is associated with β-cardiac myosin hypercontractility and impaired myocardial compliance [4, 5]. The hypercontractility often results in cardiac hypertrophy and diastolic dysfunction. Variable clinical symptoms of HCM may be associated with the severity of mitral regurgitation and diastolic dysfunction [4]. Dyspnea is one of the most common presenting symptoms [5]. HCM often progresses to obstruction of the left ventricular outflow tract (LVOT), patients may have increased dizziness, fainting or near fainting, syncope, angina, paroxysmal nocturnal dyspnoea, congestive heart failure, and sudden cardiac death [5].

Traditional medication treatments of HCM are not disease-specific, HCM medical therapy depends on the patient’s clinical picture and remains limited to beta adrenergic blockers, calcium channel blockers, angiotensin-converting enzyme inhibitors/angiotensin receptor blockers, antiarrhythmics, diuretics and oral anticoagulants [5, 6].

Mavacamten (MYK-461), a selective and reversible cardiac myosin inhibitor, is a novel β-cardiac-specific small molecule drug design-based on current knowledge of HCM disease pathology [5, 7, 8]. Mavacamten directly inhibits cardiac myosin adesonine triphosphatase (ATPase) and decreases the number of myosin heads that can enter the on-actin state, inhibiting the binding of myosin to actin and the sarcomere force and ultimately causing a reduction in the cardiac contractility and improvement in compliance [5, 8]. Up to now, results from several recent randomized clinical trials (RCTs) with mavacamten indicated that mavacamten has a favorable therapeutic potential [8].

This study aimed to perform a meta-analysis to assess the efficacy and safety of mavacamten in treating HCM from available RCTs. By synthesizing the results of all published RCTs, the efficacy and safety of mavacamten in treating HCM can be more certain. This meta-analysis will also provide guidance and direction for future research on mavacamten and its role in HCM.

## 2. Method

This meta-analysis was conducted according to the Preferred Reporting Items for Systematic Review and Meta-analysis (PRISMA) and Cochrane Handbook [9, 10]. We registered the review prospectively in Open Science Frameworks, https://osf.io/qryds/.

### 2.1 Search Strategy

PubMed, Cochrane Library, Embase and Clinical Trials were searched from inception to February 6, 2024, without language restrictions. The detailed search strategy of all databases can be seen in **Supplementary Text S1**. Searched terms were “mavacamten”, “MYC-461”, “camzyos”, “hypertrophic cardiomyopathy”, “hypertrophic obstructive cardiomyopathy”, “familial hypertrophic cardiomyopathy”, “HCM”, “HOCM”. We searched all published papers related to mavacamten, including systematic reviews and meta-analysis.

### 2.2 Eligibility Criteria

We included human studies in this study if they met the following eligibility criteria: (1) the study was an RCTs; (2) patients in the included studies were diagnosed with HCM; (3) the intervention group received mavacamten and the control group received placebo; (4) the studies reported at least one of the outcomes of interest.

If the study was a review, case reports, non-RCTs, letter, abstracts and articles with insufficient data, it was excluded

### 2.3 Study Selection

All study search records were imported into the Rayyan software (Qatar Computer Research Institute, https://www.rayyan.ai/). Two authors (YMC and XTG) independently screened the papers and accessed all articles based on the title and abstract. When there was uncertainty about whether a study met the inclusion criteria, the same two authors read the full text to confirm the relevance. Disagreements were resolved by a third author (DPL) via discussion.

### 2.4 Data Extraction

Two authors (LZ and XTG) independently extracted the data into a spreadsheet for analysis. Any discrepancies were resolved by discussion. The following data were extracted from eligible studies: first author’s last name, publication time, research design, duration of intervention, patient baseline characteristics, improvement in ≥1 New York Heart Association (NYHA) class, the Clinical Summary Score of the Kansas City Cardiomyopathy Questionnaire (KCCQ-CSS), composite functional endpoint (it was composite to assess clinical response at the last week of treatment compared with baseline, defined as at least 1.5 mL/kg/min improvement in pVO2 and a reduction of ≥ NYHA functional class; or at least 3.0 mL/kg/min improvement in pVO2 with no worsening in NYHA class), change in N-terminal pro-B-type natriuretic peptide (△NT-proBNP), change from baseline in cardiac troponin I (△cTnI), Valsalva LVOT gradient, change in peak oxygen uptake (△pVO2), ≥1 total emergent adverse event (TEAE) such as palpitations, dizziness, chest pain, etc, and ≥1 serious adverse event (SAE) such as atrial fibrillation, renal failure, infection, etc.

### 2.5 Quality of Assessment

Two authors (LZ and YMC) independently assessed the risk of bias of the included RCTs using the Cochrane Risk of Bias Tool (RoB2) [11]. Any discrepancies were resolved by mutual consensus or adjudicated by a third reviewer (DPL).

### 2.6 Statistical Analysis

This meta-analysis was performed by Review Manager (RevMan) 5.3 (Nordic Cochrane Centre, Copenhagen, Denmark). Dichotomous outcomes were assessed by risk ratios (RR) while the mean difference (MD) was employed to express the continuous outcomes data, with 95% confidence intervals (CI). We used *I*^2^ to assess heterogeneity between included RCTs, with an *I*^2^ of greater than 50% indicating at least moderate heterogeneity. Based on the assumption of considerable clinical heterogeneity, the random-effects model was used for this meta-analysis.

## 3. Results

### 3.1 Study Selection

**Fig. 1.**
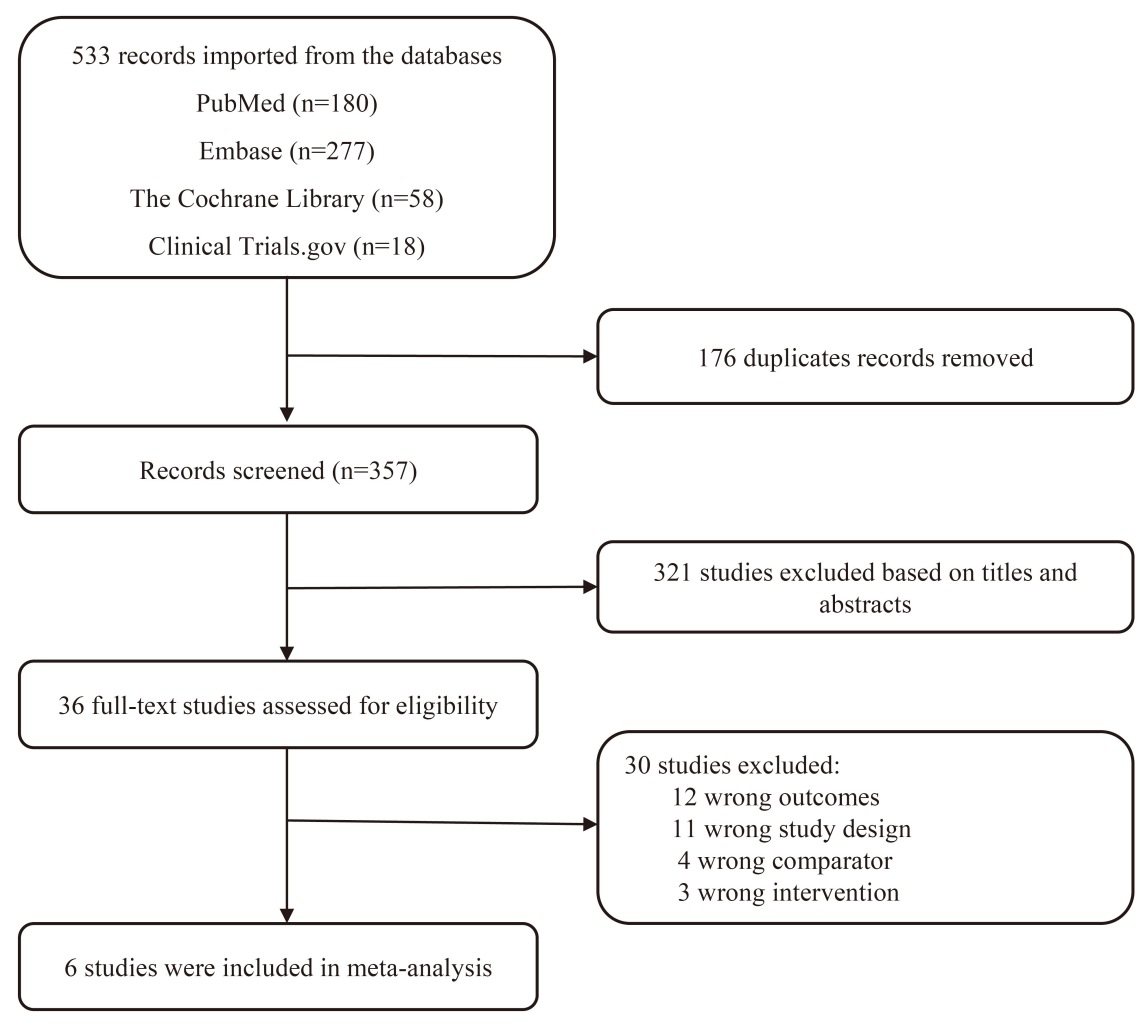
**Preferred Reporting Items for Systematic Review and Meta-analysis (PRISMA) flowchart of the study screen**.

A total of 533 studies were obtained from the above-mentioned database. Among them, 176 articles were deleted due to duplication and 321 studies were excluded based on titles and abstracts. At last, 6 studies met the inclusion criteria (Fig. 1).

### 3.2 Study Characteristics

The 6 included RCTs [12, 13, 14, 15, 16, 17] were comprised of 732 patients with HCM, of whom 388 received mavacamten and 344 received placebo. Five of the RCTs [13, 14, 15, 16, 17] were performed in obstructive HCM and one RCT [12] was performed in non-obstructive HCM. The mean age of patients was above 40 years old in these included RCTs. In this meta-analysis, most baseline characteristics were similar in either group. Table 1 (Ref. [12, 13, 14, 15, 16, 17]) presented more characteristics of the included RCTs.

**Table 1.** **The detail characteristics of the included studies**.

Study	Research design	Patients type	Interventions	No.	Average age (y)	Dose of medication	Duration of treatment (weeks)
Ho *et al*. 2020 [12]	a multicenter, double-blind, placebo-controlled, dose-ranging phase II study	non-obstructive hypertrophic cardiomyopathy	mavacamten	40	54.0 ± 14.6	200 or 500 ng/mL once a day	16
			placebo	19	53.8 ± 18.2	placebo	
Saberi *et al*. 2021 [13]	a randomized, double-blind, placebo-controlled, phase III trial	obstructive hypertrophic cardiomyopathy	mavacamten	17	60.3	2.5, 5, 10, or 15 mg once a day	30
			placebo	18		placebo	
Olivotto *et al*. 2020 [14]	a randomized, double-blind, placebo-controlled, phase III trial	obstructive hypertrophic cardiomyopathy	mavacamten	123	58.5 ± 12.2	2.5, 5, 10, or 15 mg once a day	30
			placebo	128	58.5 ± 11.8	placebo	
Spertus *et al*. 2021 [15]	a randomized, double-blind, placebo-controlled, phase III trial	obstructive hypertrophic cardiomyopathy	mavacamten	98	57.8 ± 12.7	15 mg/d	30
			placebo	96	58.2 ± 11.6	placebo	
Desai *et al*. 2022 [16]	a multicenter, randomized, double-blind, placebo controlled, phase III trial	severe obstructive hypertrophic cardiomyopathy	mavacamten	56	59.8 ± 14.2	2.5, 5, 10, or 15 mg once a day	16
			placebo	56	60.9 ± 10.5	placebo	
Tian *et al*. 2023 [17]	randomized, double-blind, placebo-controlled, phase III trial	obstructive hypertrophic cardiomyopathy	mavacamten	54	52.4 ± 12.1	1, 2.5, 5, 10, or 15 mg once a day	30
			placebo	27	51.0 ± 11.8	placebo	

### 3.3 Risk of Bias

Table 2 (Ref. [12, 13, 14, 15, 16, 17]) showed the risk of bias of the RCTs in this meta-analysis. 1 included study [12] was judged at probably low risk of bias. Overall, the quality of the included RCTs in this meta-analysis was high and illustrated a low risk of bias.

**Table 2.** **The risk of bias assessment in included RCTs**.

Study	Bias arising from the randomization process	Bias due to deviations from the intended intervention	Bias due to missing outcome data	Bias in measurement of the outcome	Bias in selection of the reported results	Other risk of bias	Overall judgement
Ho *et al*. 2020 [12]	Low	Probably Low	Low	Low	Probably Low	Probably Low	Probably Low
Saberi *et al*. 2021 [13]	Low	Low	Low	Low	Probably Low	Low	Low
Olivotto *et al*. 2020 [14]	Low	Low	Low	Low	Probably Low	Low	Low
Spertus *et al*. 2021 [15]	Low	Low	Low	Low	Probably Low	Low	Low
Desai *et al*. 2022 [16]	Low	Low	Low	Low	Probably Low	Low	Low
Tian *et al*. 2023 [17]	Low	Low	Low	Low	Probably Low	Low	Low

RCTs, randomized clinical trials.

### 3.4 Meta-analysis

#### 3.4.1 Efficacy of Mavacamten

**Fig. 2.**
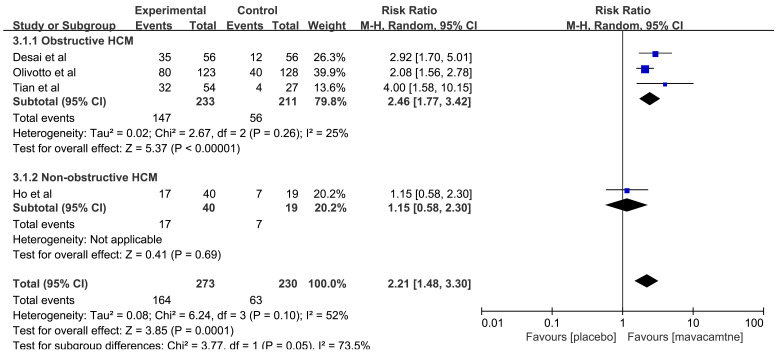
**Forest plot for improvement ≥1 New York Heart Association (NYHA) class, including two subgroups: obstructive hypertrophic cardiomyopathy (HCM) and non-obstructive HCM**. M-H, Mantel Haenszel method.

4 RCTs [12, 14, 16, 17] reported the improvement of ≥1 NYHA class. The pooled results showed a significant improvement with mavacamten (RR: 2.21, 95% CI: 1.48 to 3.30, *p* = 0.0001, Fig. 2). In the subgroup analysis for obstructive HCM, the results showed that the improvement ≥1 NYHA class was higher in the mavacamten group (RR: 2.46, 95% CI: 1.77 to 3.42, *p* < 0.00001), but there was no significant difference in the improvement ≥1 NYHA class between the mavacamten and placebo groups (RR: 1.15, 95% CI: 0.58 to 2.30, *p* = 0.69) among non-obstructive HCM.

**Fig. 3.**
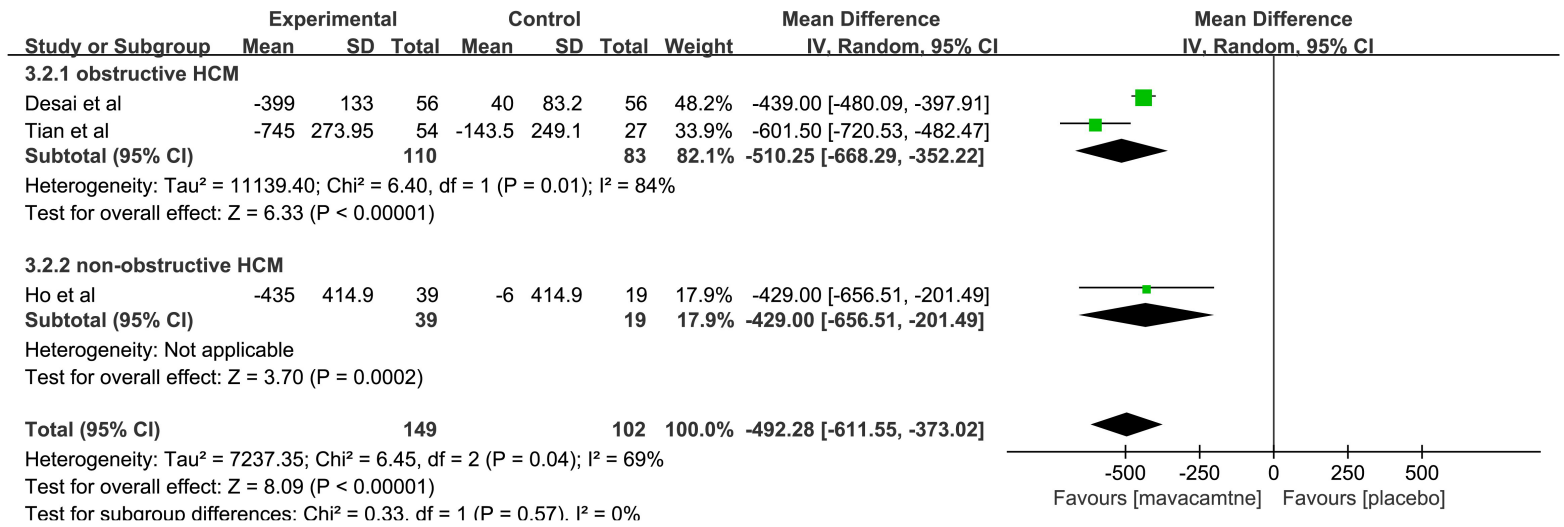
**Forest plot for change in N-terminal pro-B-type natriuretic peptide (△NT-proBNP), including two subgroups: obstructive hypertrophic cardiomyopathy (HCM) and non-obstructive HCM**. IV, inverse variance method.

3 RCTs [12, 16, 17] reported the effect of mavacamten on NT-proBNP. Two RCTs [16, 17] compared the changes in NT-proBNP from the baseline (△NT-proBNP) between mavacamten and placebo-treated obstructive HCM, and 1 RCT [12] reported non-obstructive HCM. The pooled results showed that mavacamten can significantly reduce NT-proBNP levels compared to the placebo group (MD: –492.28, 95% CI: –611.55 to –373.02, *p* < 0.00001, Fig. 3). In the subgroup analysis for obstructive HCM, △NT-proBNP in the mavacamten group was higher than that in the placebo group (MD: –510.25, 95% CI: –668.29 to –352.22, *p* < 0.00001). Meanwhile, in non-obstructive HCM, MD was changed by –429 ng/L (95% CI: –656.51 to –201.49, *p* = 0.0002) compared to the placebo group.

**Fig. 4.**
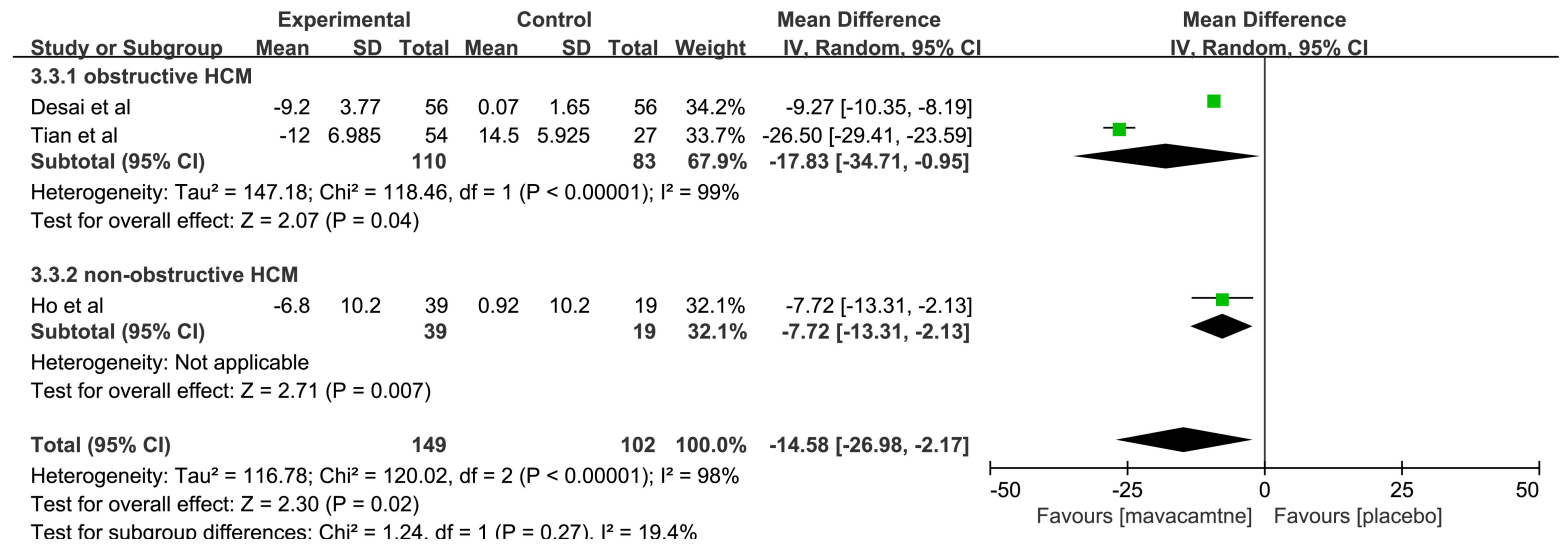
**Forest plot for change from baseline in cardiac troponin I (△cTnI), including two subgroups: obstructive hypertrophic cardiomyopathy (HCM) and non-obstructive HCM**. IV, inverse variance method.

△cTnI was evaluated in three RCTs [12, 16, 17]. Two RCTs [16, 17] reported obstructive HCM patients, and another RCT [12] was on patients with non-obstructive HCM. The pooled results showed that mavacamten can significantly reduce cTnI levels compared to the placebo group (MD: –14.58, 95% CI: –26.98 to –2.17, *p* = 0.02, Fig. 4). In the subgroup analysis for obstructive HCM, △cTnI in the mavacamten group was higher than that in the placebo group (MD: –17.83, 95% CI: –34.71 to –0.95, *p* = 0.04). Meanwhile, in non-obstructive HCM, MD was changed by –7.72 ng/L (95% CI: –13.31 to –2.13, *p* = 0.007*)* compared to the placebo group.

**Fig. 5.**
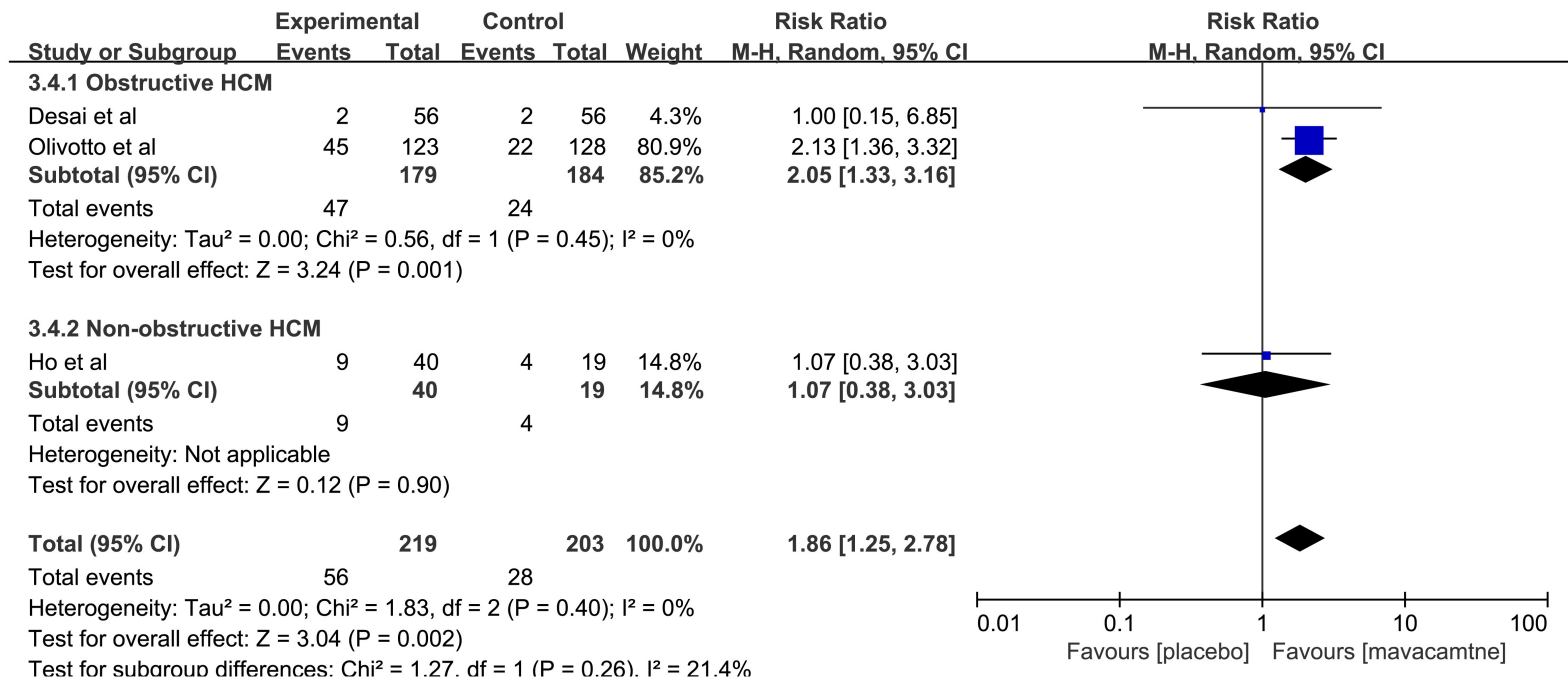
**Forest plot for composite functional end point, including two subgroups: obstructive hypertrophic cardiomyopathy (HCM) and non-obstructive HCM**. M-H, Mantel Haenszel method.

The aggregated results [12, 14, 16] showed that mavacamten can significantly improve the composite functional end points (RR: 1.86, 95% CI: 1.25 to 2.78, *p* = 0.002, Fig. 5). The subgroup results showed no significant difference in the composite functional end points among the non-obstructive HCM (RR: 1.07, 95% CI: 0.38 to 3.03, *p* = 0.90). However, in obstructive HCM, mavacamten improved the composite functional end points higher than that in placebo (RR: 2.05, 95% CI: 1.33 to 3.16, *p* = 0.001).

**Fig. 6.**
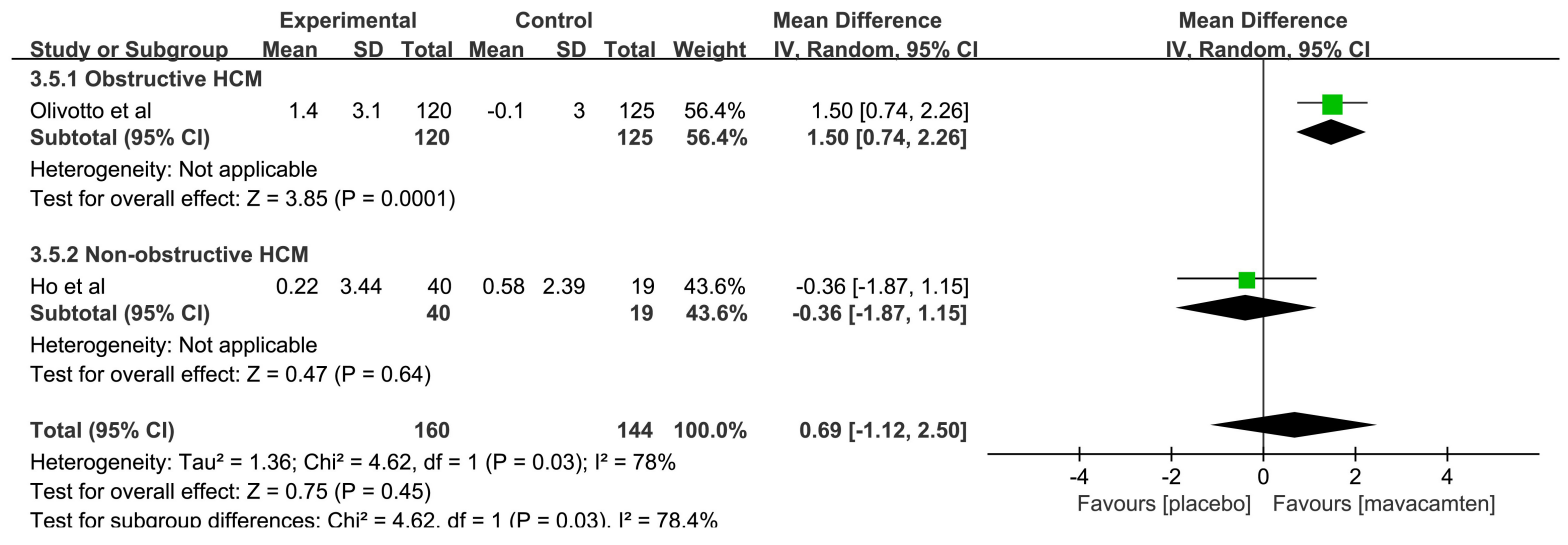
**Forest plot for peak oxygen uptake (pVO2), including two subgroups: obstructive hypertrophic cardiomyopathy (HCM) and non-obstructive HCM**. IV, inverse variance method.

△pVO2 was evaluated in two RCTs [12, 14]. 1 RCT reported obstructive HCM patients, and another RCT was on patients with non-obstructive HCM. The pooled results revealed that there was no statistically significant difference in △pVO2 between groups (MD: 0.69, 95% CI: –1.12 to 2.50, *p* = 0.45, Fig. 6). Additionally, the subgroup analysis showed that △pVO2 in the mavacamten group was higher than that in placebo group among obstructive HCM (MD: 1.50, 95% CI: 0.74 to 2.26, *p* = 0.0001), but there was no significant difference in △pVO2 between groups (MD: –0.36, 95% CI: –1.87 to 1.15, *p* = 0.64) among non-obstructive HCM.

**Fig. 7.**
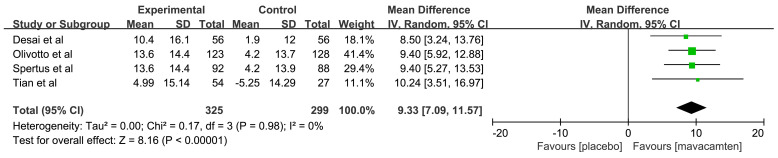
**Forest plot for Clinical Summary Score of the Kansas City Cardiomyopathy Questionnaire (KCCQ-CSS)**. IV, inverse variance method.

In the included RCTs reporting KCCQ-CSS score, all patients involved were obstructive HCM. The combined data [14, 15, 16, 17] for change from baseline in KCCQ-CSS score showed a significant improvement with mavacamten (MD: 9.33, 95% CI: 7.09 to 11.57, *p* < 0.00001, Fig. 7).

**Fig. 8.**
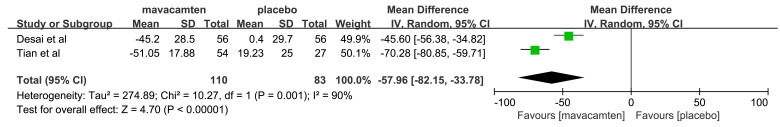
**Forest plot for Valsalva left ventricular outflow tract (LVOT) gradient**. IV, inverse variance method.

In the included RCTs reporting the change in LVOT gradient induced by Valsalva from baseline, all patients involved had obstructive HCM. With respect to the change in LVOT gradient induced by Valsalva from baseline [16, 17], a significant relationship was illustrated when mavacamten was compared with placebo (MD: –57.96, 95% CI: –82.15 to –33.78, *p* < 0.00001, Fig. 8).

#### 3.4.2 Safety of Mavacamten

4 RCTs [12, 14, 16, 17] reported the data of patients with ≥1 TEAE and ≥1 SAE. 3 RCTs investigated the incidence of ≥1 TEAE and ≥1 SAE in mavacamten and placebo-treated obstructive HCM, and 1 RCT compared the incidence of ≥1 TEAE and ≥1 SAE in mavacamten and placebo-treated non-obstructive HCM.

**Fig. 9.**
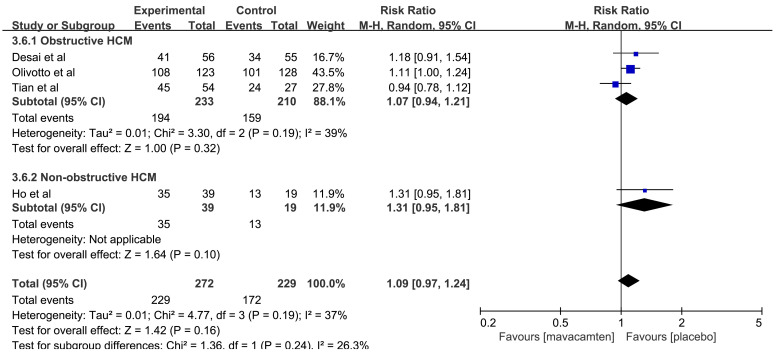
**Forest plot of patients with ≥1 total emergent adverse event (TEAE), including two subgroups: obstructive hypertrophic cardiomyopathy (HCM) and non-obstructive HCM**. M-H, Mantel Haenszel method.

**Fig. 10.**
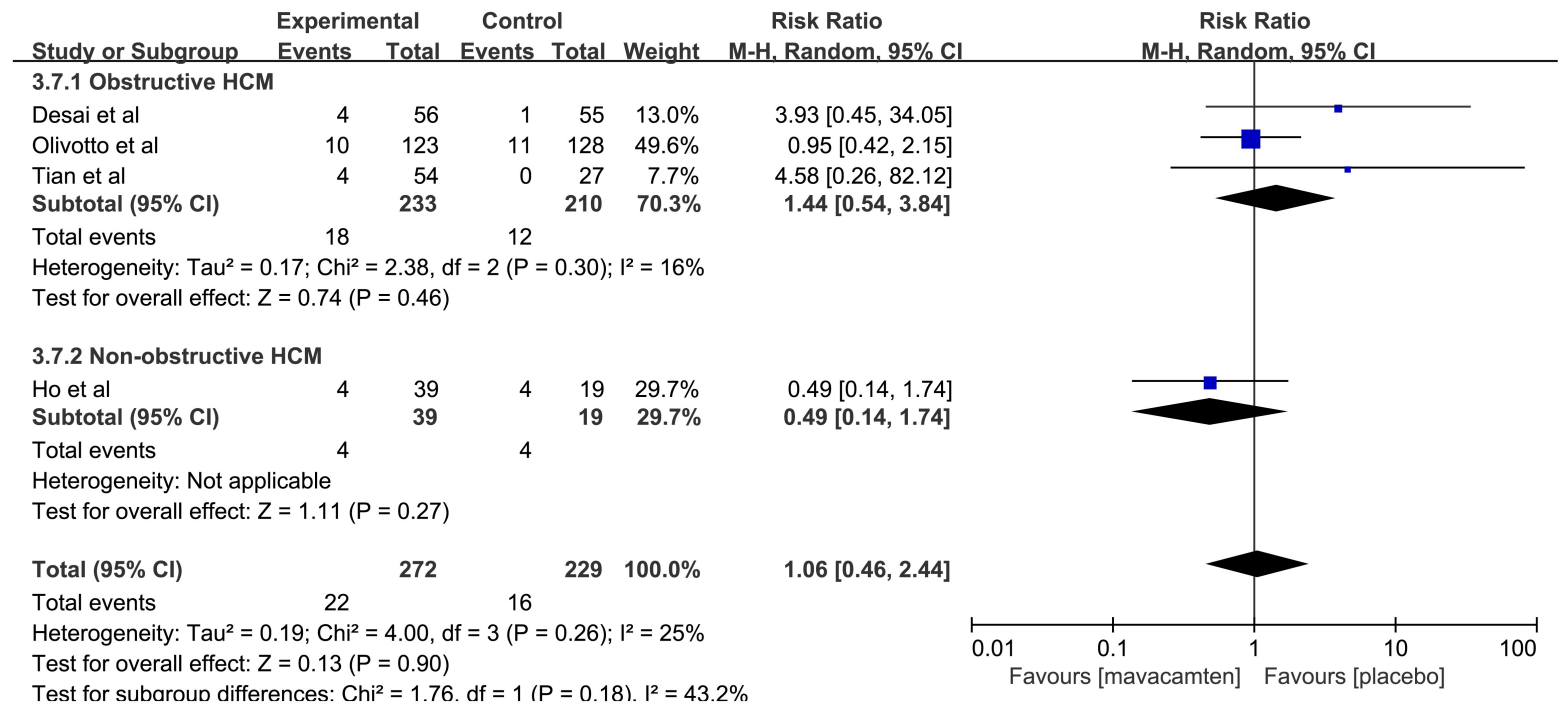
**Forest plot of patients with ≥1 serious adverse event (SAE), including two subgroups: obstructive hypertrophic cardiomyopathy (HCM) and non-obstructive HCM**. M-H, Mantel Haenszel method.

The pooled results showed that there was no significant difference with the rates of ≥1 TEAE (RR: 1.09, 95% CI: 0.97 to 1.24, *p* = 0.16, Fig. 9) between both groups. Meanwhile, the aggregated results [12, 14, 16, 17] for ≥1 SAE showed that there was no significant difference between the mavacamten and placebo groups (RR: 1.06, 95% CI: 0.46 to 2.44, *p* = 0.90, Fig. 10).

Several symptoms of TEAEs and SAEs were analyzed in this study, including palpitations, atrial fibrillation, dizziness, chest pain, infection, renal failure and so on. The detailed results are shown in Table 3.

**Table 3.** **The meta analysis of TEAEs and SAEs reported in the included RCTs**.

Type	Symptom	No. of studies (n)	Mavacamten group, n/n	Placebo group, n/n	Heterogeneity	RR	95% CI	*p*
TEAEs	Palpitations	2	8/95	5/74	I^2^ = 0%	0.97	0.34–2.82	0.97
	Atrial fibrillation	2	5/95	1/74	I^2^ = 0%	2.23	0.38–13.13	0.38
	Syncope	2	3/67	1/75	I^2^ = 0%	3.39	0.53–21.65	0.20
	Fatigue	1	5/39	3/19	NA	1.81	0.22–3.05	0.76
	Dizziness	1	7/39	1/19	NA	3.41	0.45–25.77	0.23
	Chest pain	1	2/56	3/55	NA	0.65	0.11–3.77	0.64
SAEs	Atrial fibrillation	4	8/272	5/229	I^2^ = 0%	1.06	0.34–3.33	0.92
	Infection	2	2/179	2/183	I^2^ = 0%	0.97	0.14–6.56	0.98
	Renal failure	1	1/39	0/19	NA	1.50	0.06–35.19	0.80

RR, risk ratios; CI, confidence interval; TEAEs, total emergent adverse events; SAEs, serious adverse events; RCTs, randomized controlled trials; NA, not applicable.

## 4. Discussion

6 RCTs [12, 13, 14, 15, 16, 17] allocating 732 patients diagnosed with HCM were included in this meta-analysis. Among them, 673 patients were diagnosed with obstructive HCM, and 59 patients were diagnosed with non-obstructive HCM. The results of this study provide evidence for the efficacy and safety of mavacamten for treating HCM. The main finding as follows: (1) overall, mavacamten can achieve higher rates of ≥1 NYHA improvement, KCCQ-CSS and primary composite endpoint compared to placebo; (2) NT-proBNP and cTnI were significantly lower in the mavacamten group than in the placebo group; (3) patients receiving mavacamten achieved a Valsalva LVOT gradient less than placebo; (4) mavacamten had little effect on △pVO2, as there were no significant differences in the levels of these parameters between mavacamten and placebo group among HCM patients; (5) there was no significant difference in the incidences of ≥1 TEAE and ≥1 SAE between groups; (6) the results of common symptoms of TEAEs and SAEs showed that there was no significant difference between the mavacamten group and placebo group; (7) mavacamten can significantly improve in ≥1 NYHA improvement and composite functional endpoint levels among obstructive HCM. There were no significant differences in ≥1 NYHA improvement and composite functional endpoint levels among non-obstructive HCM; (8) only two RCTs reported △pVO2. Notably, the two RCTs evaluated obstructive and non-obstructive HCM, respectively. The subgroup results showed that mavacamten can significantly improve △pVO2 levels among obstructive HCM, but there were no significant differences in △pVO2 levels among non-obstructive HCM. Therefore, when using mavacamten in patients to improve pVO2 levels, it is necessary to distinguish the type of HCM before deciding whether to use it.

During our search in the above-mentioned databases, we found several meta-analyses [8, 18, 19] have reported the effect and safety of mavacamten in the treatment of HCM. The results of our study are consistent with the conclusions stated in these 3 studies that mavacamten can effectively improve ≥1 NYHA class. However, our conclusions on the outcome indicators were different from those in the reports after we increased our sample size. Several meta-analyses [8, 18] reported that mavacamten treatment has a higher incidence of ≥1 TEAE than placebo. But after we increased our sample size, we found there was no significant difference between mavacamten and placebo in the incidences of ≥1 TEAE.

Before the emergence of mavacamten, the treatment of HCM could only be achieved through non-specific drugs and surgery to improve symptoms and prevent sudden death. But, these therapies did not show any benefits in clinical trials [20]. The emergence of mavacamten has reversed this passive situation. Mavacamten can target and affect myosin, leading to a decrease in ATPase activity [21]. While improving the symptoms and signs of HCM, it can also prevent further disease progression, reduce ventricular wall tension, improve cardiac structure, and reduce heart damage [12, 14]. Mavacamten is a major advancement in HCM precision therapy, opening a new era of HCM treatment.

Due to the limitations of conventional drug therapy for HCM patients, the emergence of a targeted drug named mavacamten is undoubtedly a ray of hope in the darkness for HCM patients. However, as a cardiac myosin inhibitor, mavacamten directly interferes with the contractile function of myocardial cells and disturbs the processes involving energy production, storage, and utilization within the myocardial cells. It also disrupts the uptake, release, and reuptake of calcium ions within the myocardial cells, thereby affecting the excitation-contraction coupling of the myocardium. This ultimately leads to a decrease in myocardial contractility and relaxation of the heart muscle. While it is effective in treating HCM, it can also result in a reduction in left ventricular ejection fraction (LVEF). It is worth noting that the specific pathological mechanism leading to a reduction in LVEF caused by mavacamten is currently not fully understood. Although the mechanism of mavacamten has been described, the specific cellular and molecular-level pathological changes have not been completely elucidated. Fortunately, several RCT [12, 14, 16, 17] findings have indicated that the LVEF reduction caused by mavacamten is reversible, and the incidence rate is low. Out of the 6 RCTs included in this study, 3 RCTs reported the effect of mavacamten on LVEF. Ho *et al*. [12] found that 5 cases of patients experienced a decrease in LVEF to below 45% after taking mavacamten, but the LVEF recovered to above 50% after discontinuation of the drug. Olivotto *et al*. [14] found that a total of 9 patients experienced a decrease in LVEF after taking mavacamten. Among them, 5 patients had their LVEF recovered to baseline levels after discontinuing mavacamten treatment, 3 patients had their LVEF restored during the drug washout period at the end of the trial, but 1 patient with concomitant atrial fibrillation opted for atrial fibrillation ablation due to severe LVEF reduction and still had incomplete LVEF recovery at the end of the trial. The research findings by Desai *et al*. [16] showed that the resting LVEF remained stable throughout the entire treatment process, with only 2 patients experiencing a decrease in LVEF after taking mavacamten. These 2 patients resumed treatment without further adverse effects and remain in the long-term extension study. Tian *et al*. [17] found that no patient had an LVEF less than 50% or developed heart failure. The above results suggest that mavacamten is generally well-tolerated, and in the few cases where there was a decrease in LVEF, most patients were able to recover to normal levels after discontinuing the medication. But the instructions for mavacamten (CAMZYOS) state two precautions: (1) Initiation of CAMZYOS in patients with LVEF <55% is not recommended; (2) Interrupt CAMZYOS if LVEF is <50% at any visit or if the patient experiences heart failure symptoms or worsening clinical status. These should draw the attention of general cardiologists/clinicians.

The Food and Drug Administration (FDA) has approved mavacamten for the treatment of HCM, which can further expand the sample size of clinical studies and obtain more reliable data based on current clinical studies. Meanwhile, different from the race and living environment limitations of foreign clinical research samples, RCTs of mavacamten should be conducted on a large scale in the Asian region. Additionally, there are several aspects that need to be further explored regarding the research of mavacamten: (1) conduct clinical trial research with the main indicator of “hard endpoint”, such as reducing death, atrial fibrillation, and heart failure; (2) conduct clinical research on the correlation between mavacamten and the occurrence of arrhythmia; (3) conduct research on contraindications of mavacamten; (4) conduct research on interactions between mavacamten and other therapeutic drugs; (5) although there is no difference in the incidence of TEAE and SAEs between the mavacamten group and the placebo group in this study, the longest follow-up time of the included RCTs was only 30 weeks, and long-term monitoring is still needed in the future, which requires real-world observation and follow-up after marketing.

Compared with other meta-analyses [8, 18, 19], we have expanded the sample size and obtained results that are more informative, but our study still has several limitations. First, only 6 RCTs met our inclusion criteria as mavacamten is a new drug in the market. As a result, these findings may underestimate the efficacy and overestimate the safety of mavacamten. More RCTs with a large number of patients are needed to obtain a definite conclusion. Second, not all included RCTs reported the outcome indicators for evaluation, which further restricted the sample size for certain outcomes. Third, the longest follow-up time of the included RCTs was only 30 weeks, hence the value in predicting clinical effects is limited, and we have to rely on post-marketing safety surveillance systems for adverse events (AEs). Long term research data is needed to confirm the safety and efficacy of mavacamten. Nonetheless, our findings still provide preliminary evidence supporting favorable outcomes of mavacamten in treating HCM.

## 5. Conclusions

This meta-analysis found that mavacamten could improve the NYHA function class, KCCQ-CSS scores and composite functional end point. Meanwhile, mavacamten can decrease the NT-proBNP, cTnI and Valsalva LVOT gradient. This meta-analysis did not find any increased risk of AEs or SAEs following treatment with mavacamten. Further research should focus on a long-term follow up study to evaluate the efficacy and safety of mavacamten for the treatment of HCM.

## Availability of Data and Materials

The data presented in the study were included in the article or in its supplementary material.

## Supplementary Material

Supplementary material associated with this article can be found, in the online version, at https://doi.org/10.31083/j.rcm2510375.
