# Supplementary material for: A Systematic Review and Meta-analysis of Efficacy and Safety of Mavacamten for the Treatment of Hypertrophic Cardiomyopathy
Source: Rev Cardiovasc Med. 2024 Oct 23;25(10):375. doi: 10.31083/j.rcm2510375 (PMC11522755; doi:10.31083/j.rcm2510375)
Supplement: Supplementary file 1 [file 2153-8174-25-10-375-s1.zip › Text S1.docx]

**Text S1**

**PubMed:**

#1 hypertrophic cardiomyopathy*[Title/Abstract]

#2 "mavacamten"[Supplementary Concept]

#3 "mavacamten"[Title/Abstract] OR "MYK-461"[Title/Abstract] OR "SAR439152"[Title/Abstract] OR "camzyos"[Title/Abstract]

#4 #2 OR #3

#5 "Randomized Controlled Trials as Topic"[Mesh] OR "Randomized Controlled Trial"[Publication Type] OR "Equivalence Trial"[Publication Type] OR "Pragmatic Clinical Trial"[Publication Type] OR "Equivalence Trials as Topic"[Mesh] OR "Intention to Treat Analysis"[Mesh] OR "Pragmatic Clinical Trials as Topic"[Mesh] OR "Single-Blind Method"[Mesh] OR "Random Allocation"[Mesh] OR "Double-Blind Method"[Mesh] OR "Random Allocation"[Mesh] OR "Adaptive Clinical Trial" [Publication Type] OR "Adaptive Clinical Trials as Topic"[Mesh] OR "Clinical Trials, Phase II as Topic"[Mesh] OR "Clinical Trials, Phase III as Topic"[Mesh] OR "Clinical Trials, Phase IV as Topic"[Mesh]

#6 random*[Title/Abstract] OR 'equivalence trial'[Title/Abstract] OR 'equivalence clinical trial'[Title/Abstract] OR 'equivalence design'[Title/Abstract] OR 'non-inferiority trial'[Title/Abstract] OR 'noninferiority trial'[Title/Abstract] OR 'pragmatic trial'[Title/Abstract] OR 'non-inferiority clinical trial'[Title/Abstract] OR 'non-inferiority design'[Title/Abstract] OR 'practical clinical trial'[Title/Abstract] OR 'pragmatic clinical trial'[Title/Abstract] OR 'superiority clinical trial'[Title/Abstract] OR 'superiority design'[Title/Abstract] OR 'superiority trial'[Title/Abstract] OR 'single masked'[Title/Abstract] OR 'single blind'[Title/Abstract] OR 'single-blind'[Title/Abstract] OR 'double masked'[Title/Abstract] OR 'double blind'[Title/Abstract] OR 'double-blind'[Title/Abstract] OR 'triple masked'[Title/Abstract] OR 'triple blind'[Title/Abstract] OR 'triple-blind'[Title/Abstract] OR singleblind*[Title/Abstract] OR doubleblind*[Title/Abstract] OR tripleblind*[Title/Abstract] OR 'intent to treat'[Title/Abstract] OR 'intention to treat'[Title/Abstract] OR 'adaptive design'[Title/Abstract] OR 'adaptive trial'[Title/Abstract]

#7 #5 OR #6

#8 #1 AND #4 AND #7

**Embase:**

#1 'randomized controlled trial'/exp OR 'controlled clinical trial'/exp OR 'randomization'/exp OR 'randomized':ab,ti OR 'randomised':ab,ti OR 'randomly':ab,ti OR 'clinical trial (topic)'/exp OR 'placebo'/exp OR 'placebo':ab,ti OR 'trial':ab,ti

#2 'animal'/exp NOT 'human'/exp

#3 #1 NOT #2

#4 'mavacamten'/exp OR 'MYK-461':ab,ti OR 'SAR439152':ab,ti OR 'camzyos'

#5 #3 AND #4

#6 hypertrophic cardiomyopathy*:ab,ti

#7 #5 AND #6

**Cochrane Central Register of Controlled Trials:**

#1 MeSH descriptor: [hypertrophic cardiomyopathy] explode all trees

#2 ("mavacamten"):ti,ab,kw OR ("MYK-461"):ti,ab,kw OR ("SAR439152"):ti,ab,kw OR ("camzyos"):ti,ab,kw

#3 #1 AND #2

**Clinical Trials.gov:**

Condition or disease: hypertrophic cardiomyopathy

Intervention/Treatment: mavacamten or MYK-461 or SAR439152 or camzyos
